# On the Moral Phenomena of Insanity and Eccentricity

**Published:** 1861-04

**Authors:** Thomas Mayo


MEDICAL CRITIC

AND

PSYCHOLOGICAL

JOURNAL.

APRIL, 1861.

### Art. I.—ON THE MORAL PHENOMENA OF INSANITY AND ECCENTRICITY.

By Thomas Mayo.

A state, which may seem to deserve the name of Moral Insanity, as exhibiting a perversion of the moral sentiments, tendencies, and perceptions, with no slight loss of self-control, must be recognised as often prominent in the early stage of mental disease, and before the intellect is palpably affected. When certain delusions, when delirium or incoherency supervene, the case obtains without question the name of insanity. While most cases begin in this way, a very palpable difference of a practical kind is made by many reasoners in nomenclature; some extending the epithet insane to all those who exhibit these moral phenomena, whether combined with intellectual perversion or not; others refusing to assign it, unless the intellectual lesion be also patent in the case. Up to this point in the history of mental affection the patient must be held, in their opinion, personally responsible for his conduct in a criminal sense; while, with those who are disposed to give moral phenomena an equal weight as pathognomonic of insanity with those of the intellect, the moral phenomena which, with the former, are only recognised as having been insane, when an intellectual aberration has also occurred, are at once recognised as possessing an independent right to constitute a lunatic.

The grounds on which an intellectual as well as a moral aberration are deemed necessary, where insanity is presumed to confer irresponsibility in regard to crime, appear to me good. I have seen no reason to question the importance of this rule, which certainly tends to maintain the boundaries of vice and madness,—so that a murderer should not escape justice on this kind of plea, unless he had superadded to the phenomena of moral disorder those of intellectual disorder ; the assumption which underlies this argument being that so long as his intellect is unperverted, he will be found to possess a consciousness of the nature of the criminal act in relation to law. This has been argued by the writer of the present essay and by others, and appears to be a prevalent doctrine with the judges. But it does not form my present object to carry it farther. I wish to guard against a certain apparent parity of reasoning which may leave both the patient and the public unprotected, should the moral symptoms of insanity obtain no recognition from the law until intellectual perversion has been recognised.

I have assumed that the patient may not with safety to society be considered legally irresponsible as mad while the moral stage, or what shall appear to be the moral stage, of the disease is alone perceptible in his motives and actions. But can the law give him no protection until then ? He may destroy the comforts of his family and ruin their fortunes and his own ; he may have become a bad father, a savage husband, a profligate and licentious member of society, and a total change of character may have occurred with these symptoms ; but no false perceptions, no amount of delirium or incoherency may have given evidence that he is mad, on the principles on which I am supposing that state to be made good in the strict meaning of the term,—here is a difficulty which must not be overlooked, in connexion with the above distinctions. In a word, I wish to establish the point, that a different practical criterion must be sought for as to what insanity means, where the case in question is one, in which the agent is claiming protection against the consequences of a crime, on the ground that he is irresponsible,—and where he and his family are claiming protection for themselves and surveillance for *him*, on the ground that he is unfit to manage his person and property. We cannot wait to clear up the question whether the definition of insanity, such as it ought to be, has been accomplished in the supposed case, so as to enable us to coerce it by a certificate of unsoundness of mind, before it has reached a Cenci *dénouement*, or such an one as Feuerbach brings forward in his work on jurisprudence, in which the lives of a whole family were saved by their concurring to put to death a homicidal father. The law will not permit the idea of insanity in the agent to plead his excuse when it knows that he is perfectly aware of the murderous tendency of his actions, and in being unable to resist them is only in the same predicament with every recognised aspirant to the gallows. On the other hand, while it refuses to him the protection of a madhouse against the consequences of his criminal acts, it will feel—certainly it ought to feel—averse to deny him the preventive protection of a madhouse, when his friends claim it for him, both for his sake and their own, before a guilty outbreak has occurred.

I have observed that the judges of the land are willing to accept the definition of insanity, which I claim as appropriate when the plea is to confer irresponsibility; and they are right; but they will cease to be right, if they do not award the privileges of insanity at a less advanced stage of it when such may be the results of restraint and coercion.

There is no subject in which the inability of language to make good *practical distinctions* is more felt than in this. It expresses the tendencies of the rules to be laid down, rather than the exact occasions for their application. Thus, when irresponsibility in criminal cases has to be conferred on the *actual* delinquent with due protection to the interests of the public, the definition of insanity in its completest form must be predicated of him; where all that is required of the law amounts to the protection of the *possible* delinquent's person and family, it would appear quite sufficient that a case should be made out of inability to control such conduct as may reasonably be expected to culminate in insanity. It must be admitted that the variety of terms assigned in the medical certificates for the use of witnesses in designating mental lesion facilitates this operation.

Thus both social and individual interests require that the moral phenomena of insanity should be permitted to justify coercion and surveillance when the moral symptoms of insanity alone are present; and such are the considerations by which it appears to me that the doctrine of moral insanity should be estimated by the law. In this point of view, it is the early period of yet imperfect insanity; and thus viewed, it is not one head of a division of which insanity is the genus, as Pinel considers it, but a state almost always recognisable in the early condition of those who eventually become insane—though not always proceeding into that development or obtaining the genuine characteristic of the formed disease, viz., the intellectual lesion. The question whether a phase of this moral perversion justifies us in leaving it under the cognate condition called eccentricity, or contains, though dimly perceived, those elements of deficient self-control which we may deem, not indeed exculpatory of criminal acts, but justificatory of our protecting the patient against himself—this question is full of difficulty. To an experienced psychologist there may be strong grounds in a given case, and that in very early life, for suspecting that a false perception underlies what he would willingly call eccentricity. How may such phenomena be distinguished from eccentricity, so that the interference of the law may not become an unjustifiable interference with liberty or an unnecessary stigma to future life? The amount of self-control possessed by the patient must be taken into the account in reference to the probability that any such morbid perception should gain the mastery over him. It will often be an important indicant that such morbid impressions underlie his eccentricity, if he is noticed to make motiveless but voluntary gesticulations; if talking to himself he is observed to be occasionally talking to some one else, some imaginary personage; apparently motiveless conduct is always suspicious. An unreasonable fancy that he is watched and noticed is the rudiment often of a deep-rooted conviction that there is a conspiracy against him—one of the most frequent maniacal fancies when the intellectual development of the disease has been reached. Meanwhile the class which I am describing is not the less under these singular influences, because they can sometimes play with them or use them with a cunning purpose. It is indeed difficult to find one's way through the intricacies of the *perverted* phenomena acting on the more *normal*. The late Dr. Warburton and I were requested by our friend, the late Dr. Monro, with his usual solicitude on behalf of his patients, to help him towards solving a doubt which he entertained respecting the *existing* state of one of his patients. The man had laboured more than once under unquestionably insane symptoms. But we ascertained that he was well aware of his state, as well as of the opinion entertained by the world in regard to such symptoms; and being a profligate and unprincipled fellow, knew how to encourage their evolution, when they were called for, by some infamous gratification or indecent *bizarrerie*, as he much preferred an establishment to a prison, which, *as a perfectly sane man*, he would have frequently incurred. He had divested himself of his abnormal symptoms to a remarkable degree when we saw him, and Dr. Monro had been urgently called on to let him out by his unfortunate wife, because on his eventual enlargement, if not then permitted, he would, she said, terribly revenge himself on her.

Doubtless, these symptoms, wavering between eccentricity and insanity, but combined with vicious propensities, are often received into an asylum, when a prison would be more appropriate. I was told lately by Mr. Pownall, Chairman, I think, of the Brentford Quarter Sessions, the following anecdote respecting Oxford, who afterwards attempted the Queen's life. Some time before that act he was brought before Mr. Pownall and another magistrate, on account of some very eccentric cruelty shown towards some fowls; and for this offence let off with a reprimand. Seeing Mr. Pownall some time afterwards, when in the penal wards of Bedlam—"Had you," said Oxford to that gentleman—"had you punished me when I was brought before you for that former offence, I should not now have been here."

In this point of view, the case of the Hon. Mr. Tuchet was probably a sad instance of mismanagement, both legal and educational. Mr. Tuchet wantonly shot the marker in a shooting gallery. Before this event, while this young gentleman was on the town in a state of progressively increasing discontent and *ennui*, if the eye of science had been brought to bear upon him, the observer might have possibly seen good reason for calculating upon his exhausting his powers of self-control so far as to acquire good grounds for claiming the protection of the law, before he had rendered his claim to that protection questionable or inappropriate by an act which, at that stage of abnormal conduct, assumed all the frightful character of murder. It is difficult, without more knowledge than we possess of the antecedents of this gentleman, to substantiate completely our hypothesis, but it may be plausibly suggested that he was protected by the decision of a court of justice from punishment for a great crime on the plea of insanity, instead of being prevented from committing that or similar crimes by early surveillance and detention. Meanwhile, the punishment which he thus escaped was *legally* deserved, as he unquestionably well knew the murderous nature of the act which he committed at the moment of commission.

We are liable to the imputation of throwing out an intricate and entangled view of a subject, of which, however, the importance must be admitted. It must be remembered that no chart at present exists to guide us through the contra-indicants which embarrass us in our attempts to reconcile punishment with justice, where some amount of unsoundness of mind is admitted to exist—and coercion with the liberty of the subject, where the power of thought, though weakened, is not abolished. Whatever is the value of the distinctions which I am endeavouring to lay down, it is a painful reflection that the applying them in practice is left to so imperfect a method as the trial by jury. Surely this is a task which better befits the judges of the land.

If in the above remarks I have maintained the opinion that insanity is incomplete as a ground of protection to delinquents, so long as its symptoms are ethical alone, and not intellectual also, I have not the less considered that it often requires to be made the subject of coercion and surveillance long before any unequivocal evidence of diseased intellect exists. This view opens out a large vista of duties belonging to the psychologist who presides over an asylum, both as to deciding when he may justly consider that its restraints, skilfully managed, will be applicable to a given case, and as to modifying the nature of those restraints and the modes of pleasure, comfort, and encouragement which the patient can bear, so that such patients may be tempted to take refuge in an asylum rather than be taken to it. In this way, and fulfilling these conditions, the proprietor of an establishment may well lay claim to a very high position among the practical philosophers of a country. The habits of mind which he thus forms may not only cure a morbid state, but develope unrecognised mental powers.

Nearly allied with these views, I may mention a very important change which is wanting in the entire education of this country. Certainly, as applied to the higher classes, it assumes as its object the regulation of character contemplated only in its normal state. The *ordinary* vices of the young obtain correction; but of the *extraordinary* and *eccentric* or *abnormal* elements of defective characters the school or college is kept ostentatiously clear. That is to say, the persons labouring under them are not treated, but expelled; and yet such persons, not deserving to be called mad, form a large element of society. I will illustrate these remarks by a few cases, with the treatment which they have appeared to suggest. I was consulted, many years ago, respecting a boy who, as he emerged out of childhood, showed a strong tendency to low company, unreasonable likes and dislikes, to what may be called general recklessness of character, and deficient sympathy with others; at the age of about thirteen he was sent to Rugby, and in a short time expelled from it, not roughly or depreciatingly, but as a case out of their department of education. But what was to happen next? It had clearly become a case for the discriminating management of a private tutor. But the private tutor, a clergyman of course, was equally worsted. A respectable farmer was next had recourse to, as likely to gratify the boy's taste for lower company than appertained to his social position, in the most creditable, or least discreditable, way. But this was turned by him to a bad account; and now sottishness and low company were closely besetting him. Consulted by his mother, I told her that the medical profession afforded to its members a larger knowledge of the human mind, than the church, the farm house, or the public school, and that this knowledge was wanted to him who should pretend to manage her son; and I promised to look out for some young member of our profession, who would undertake to travel with her son. The plan was accepted, and it answered; that is to say, a downward progress was arrested, and the subject of it was raised to a much higher pitch of moral worth and steadiness of character in which he has since remained. But a gentlemanlike tone of mind has never been reached by him.

In another case of the same kind, circumstances permitted me to adopt a much bolder plan. He was a boy, aged about seventeen, who had by that time defeated almost every system of education, and had a fair chance of bringing himself to prison or the gallows, unless certain tendencies to indecency and to violence in his character either became sufficiently marked to render him irresponsible as an undoubted maniac, or could be arrested or placed within his control. This was in the year 1831. A very excellent establishment in my neighbourhood, in which I believed he might obtain this wanting education, as well as the positive restraint which some recent outbreaks appeared to justify, on the plea of unsoundness, gave me the means of subjecting this youth to the firm and passionless surveillance which only an asylum, or a place conducted in some measure on the principle of an asylum, can afford. The proprietor of it was well known to me as a gentleman of excellent judgment and an amiable character.

I took him to this establishment, in 1831, accompanied by his father and another relative, showed him at once into his apartment, and briefly told him why he was placed there, and how inflexible he would find his restraint until he should have gained habits of self-control. At the same time I pointed out to him the beautiful and wide grounds of the establishment, and the many enjoyments which he might command by conformableness. This I stated to him, in the presence of his two relatives, whom I then at once removed from the room. When I saw him about an hour afterwards, the nearest approach that he made to surprise or regret, was the expression, that "he never was in such a lurch as this before."

For about a fortnight he conducted himself extremely well. He then lost his self command, kicked his attendant, and struck him with a bottle of medicine. On this I went over to see him. He vindicated himself with his usual ingenuity, but looked grave and somewhat frightened when I told him that, if he repeated this offence, he would be placed under mechanical restraint, not, indeed, as a punishment, but as a means of supplying his deficiency in self-control. He expresses no kindly or regretful feeling towards his relatives; but confesses the fitness of his treatment and confinement. It appears to me that he is *tranquillized* by his utter inability to resist. From this time, during his stay at the establishment, which I continued for fourteen months, no further outbreak against authority took place. He ceased to be violent, because the indulgence of violence would imply risk of inconvenience to himself, without the comfort, which he had formerly derived from it, in exciting the anger of his friends and giving them pain. His attempts at sophistry were thrown away upon us; his complaints of the hardship involved in the nature of the restraints imposed upon him, namely, the limitation to the grounds of an establishment, regular hours, and the constant presence of an attendant, were met by a calm affirmation that he had himself admitted the necessity of some control, and that he had surmounted every other form of it. I encouraged correspondence with myself; but when any one of his letters was insolent and wayward, I declined accepting the next letter until some time should have elapsed. He read much—for we supplied him with books; and I sometimes engaged him in literary conversation. Two or three times I obtained from him a tolerably well construed Latin lesson. This, however, was to him a school of moral rather than intellectual advancement. A sustained attempt at tuition would have supplied, under present circumstances, too many opportunities of irritation between the teacher and the scholar. The *temper* requisite for the reception of knowledge and the cultivation of the intellect was *being formed,* and could not safely have been *assumed.* The same consideration induced me to postpone to him the motives and sanctions of religion. It gradually became observable, both to myself and the proprietor of the asylum, that he was becoming comparatively happy. He entered freely, and with little acrimony, into conversation with us. His complaints of the injustice of his detention became formal, and assumed the character of lodging a protest rather than making a remonstrance. Sometimes he very ingenuously admitted the freedom from unhappiness which he experienced in his present state, and compared it favourably with that in which he had previously lived, always wretched himself, but occasionally enjoying the miserable comfort of making others yet more wretched. In the course of several of my interviews, I observed the valuable influence exercised upon him by the fear of becoming irregular in mind through the indulgence of intemperate violence. The establishment itself had supplied him with a few cases in point. One young man, who had struck his father, and from that time was a wretched maniac, drew his attention.

He generally dined alone. Occasionally, and by invitation, with Mr. N——'s family. He associated with some of the patients. He never made any attempt to escape from the place, in fact, he felt himself mastered, and submitted.

After he had been about a year in this place, he exhibited a trait of character which gave us pleasure. We found that he had given ten shillings to an attendant, by whom we had reason to believe that he had not been respectfully treated.

But the increasing quietness with which he adverted to, and remonstrated against his detention, most tended to assure us that we might soon bring it to a close.

The time indeed was now arriving at which it seemed reasonable to bring to a conclusion a method of treatment, which nothing could have justified in the case to which it was applied, except the extreme importance of the principle which it embodied, and the difficulty of finding any other means of carrying that principle into effect. Towards the end of the fourteenth month of his stay I obtained for my young friend, as a private tutor, a gentleman in whose family he should reside on leaving the establishment with three or four other private pupils; and I determined he should be removed thither by one of those relatives who had conveyed him to the establishment. At the private tutor's my young friend was considered gentlemanlike and companionable; if opposed and thwarted, showed no symptoms of his ancient violence; waywardness was discoverable occasionally, but was no longer a property which defied self-control. On leaving his tutor's at the end of about a year, in order to commence professional studies, he dined and slept at my house, and conducted himself in a cordial and agreeable manner.

In order that the successful issue of this case, verified as it has been by my subsequent inquiries, may not place the system under false colours, I may observe that I do not think it could have been carried out in this form but for certain points of character existing in the patient which adapted him to the treatment applied. Without possessing active courage, he had much firmness and power of endurance; and although his scanty moral principle had not given him habits of veracity, yet he possessed in a high degree the tendency to think aloud; he was naturally frank. Indeed the openness with which he would let out those thoughts, which it was most his interest to keep secret in his evil days, was in constant contrast with the perfect unfairness and disingenuousness of his arguments in support of *them* or in vindication of his conduct. Now, the firmness of his character enabled him to endure what would have shocked weak minds—the name of a madhouse; while his frankness made it impossible for him to conceal his thoughts and feelings, and thus enabled both myself and the excellent proprietor of the establishment perfectly to estimate the effect of our measures on his character while they were proceeding.

"Quis teneat vultus mutantem Protea nodo?"

In the above remarks I have endeavoured to accomplish this kind of difficulty; for I have endeavoured to discover means of identifying the moral phenomena of the insane state, as distinct from those which may be left to the expressive term eccentricity. And at the same time I have proposed to establish certain practical relations between these states through a modified application of the same principles of treatment to both.

